# Structure-Function Studies of Sponge-Derived Compounds on the Cardiac Ca_V_3.1 Channel

**DOI:** 10.3390/ijms24043429

**Published:** 2023-02-08

**Authors:** Anne-Sophie Depuydt, Piyush A. Patel, Žan Toplak, Chinmaya Bhat, Manuela Voráčová, Irene Eteläinen, Fiammetta Vitulano, Tanja Bruun, Antti Lempinen, Nives Hribernik, Eero Mäki-Lohiluoma, Louise Hendrickx, Ernesto Lopes Pinheiro-Junior, Tihomir Tomašič, Lucija Peterlin Mašič, Jari Yli-Kauhaluoma, Paula Kiuru, Jan Tytgat, Steve Peigneur

**Affiliations:** 1 Toxicology and Pharmacology, Campus Gasthuisberg, University of Leuven, Onderwijs en Navorsing 2, Herestraat 49, P.O. Box 922, 3000 Leuven, Belgium; 2 Drug Research Program, Division of Pharmaceutical Chemistry and Technology, Faculty of Pharmacy, P.O. Box 56 (Viikinkaari 5 E), University of Helsinki, FI-00014 Helsinki, Finland; 3 Faculty of Pharmacy, University of Ljubljana, Aškerčeva cesta 7, 1000 Ljubljana, Slovenia

**Keywords:** T-type calcium channels, ion channels, purpurealidin I, bromotyrosines, marine-derived bioactive compounds

## Abstract

T-type calcium (Ca_V_3) channels are involved in cardiac automaticity, development, and excitation–contraction coupling in normal cardiac myocytes. Their functional role becomes more pronounced in the process of pathological cardiac hypertrophy and heart failure. Currently, no Ca_V_3 channel inhibitors are used in clinical settings. To identify novel T-type calcium channel ligands, purpurealidin analogs were electrophysiologically investigated. These compounds are alkaloids produced as secondary metabolites by marine sponges, and they exhibit a broad range of biological activities. In this study, we identified the inhibitory effect of purpurealidin I (1) on the rat Ca_V_3.1 channel and conducted structure–activity relationship studies by characterizing the interaction of 119 purpurealidin analogs. Next, the mechanism of action of the four most potent analogs was investigated. Analogs **74**, **76**, **79**, and **99** showed a potent inhibition on the Ca_V_3.1 channel with IC_50_’s at approximately 3 μM. No shift of the activation curve could be observed, suggesting that these compounds act like a pore blocker obstructing the ion flow by binding in the pore region of the Ca_V_3.1 channel. A selectivity screening showed that these analogs are also active on hERG channels. Collectively, a new class of Ca_V_3 channel inhibitors has been discovered and the structure–function studies provide new insights into the synthetic design of drugs and the mechanism of interaction with T-type Ca_V_ channels.

## 1. Introduction

T-type calcium channels are members of the superfamily of voltage-gated calcium (Ca_V_) channels. These channels are represented by three genes that encode three different Ca_V_3 α1-subunits [1,2]. The Ca_V_3.1 (α1G) and Ca_V_3.2 (α1H) T-type Ca_V_ channel isoforms are present mainly in the heart [3]. However, it has been shown that Ca_V_3.3 (α1I) channels are also expressed in Purkinje fibers [4]. The Ca_V_3 channels are involved in cardiac automaticity, development, and excitation–contraction coupling in normal cardiac myocytes. Their functional role becomes more pronounced in the process of pathological cardiac hypertrophy and heart failure [5,6,7]. Studies have shown that Ca_V_3.1 knockout mice present with bradycardia, confirming the role of these channels in pacemaking activity [8,9]. Mice lacking Ca_V_3.2, unlike mice lacking Ca_V_3.1, showed severely suppressed pressure overload-induced hypertrophy. Furthermore, angiotensin II-induced cardiac hypertrophy was suppressed in mice deficient for Ca_V_3.2 [6,10]. Ca_V_3.3 null mice appeared rather normal [11].

The family of Ca_V_3 channels mediates a transient (or T-type) current. These channels open near-resting membrane potential due to their low activation threshold. Hence, they are classified as low-voltage-activated (LVA) channels. Moreover, they are characterized by fast inactivation and slow deactivation kinetics [2]. Given their unique biophysical properties, T-type Ca_V_ channels are ideally suited to regulating pacemaker activity, cellular excitability, and low-threshold firing [1,2]. High-voltage-activated (HVA) calcium channels include the Ca_V_1 and Ca_V_2 families, which conduct L-, P/Q-, N-, and R-type currents. In contrast to the HVA channels, co-assembly with auxiliary subunits, such as α2/δ-, β-, and γ-subunits, is not required for Ca_V_3 channels [1].

Currently, no Ca_V_3 channel inhibitors are used in clinical settings. Mibefradil is a potent T-type calcium channel blocker and is 10- to 15-fold more selective for T-type than L-type calcium channels [12,13,14]. This non-dihydropyridine molecule, which is sold under the name Posicor^®^, was previously used in the treatment of hypertension. In 1998, it had to be withdrawn from the market due to severe drug–drug interactions. Mibefradil has been repurposed as a therapeutical agent to treat solid tumors [15]. Currently, two compounds, Z944 and ACT-709478, are in phase II clinical trials for the treatment of pain and generalized epilepsy, respectively [11,16,17].

To identify novel T-type calcium channel ligands, purpurealidin analogs were electrophysiologically investigated on Ca_V_3-expressing oocytes. Purpurealidins are alkaloid-type secondary metabolites produced by marine sponges. More specifically, the synthetic compounds investigated in this study are derived from sponges from the family Aplysinellidae (Order Verongiida) [18]. These sponges are widespread and can be found mostly in tropical and temperate regions of the world. Their secondary metabolites are reported to exhibit a broad range of biological activities, such as their antiproliferative, antiangiogenic, antibiotic, cytotoxic, and hemolytic properties [19]. In the past years, the U.S. Food and Drug Administration (FDA) has already approved several sponge-derived compounds for clinical use. For example, cytarabine is on the market as an anticancer agent, and vidarabine as an antiviral agent [20,21]. When they are converted into the triphosphate form, they are reported to inhibit DNA polymerase during the S-phase of DNA replication. This caused great interest in marine sponges as a source of compounds with pharmaceutical potential. Importantly, we noticed that the structure of purpurealidin I (1) resembles the structure of Z944, which was mentioned previously.

Following the observation of structural homology between Z944 and bromotyrosine purpurealidin I (1), we started by investigating the ex vivo activity of purpurealidin I (1). This bioactive compound can be found in the marine sponge *Psammaplysilla purpurea* [22]. We used the two-electrode voltage clamp technique to investigate its activity on Ca_V_3.1 channels. We have previously reported syntheses of sets of compounds with bromotyrosine, bromotyramine, or diarylamine scaffold based on the purpurealidin I (1) structure [18,23,24,25]. These synthetic compounds are further referred to as purpurealidin analogs. In total, 119 purpurealidin analogs of this marine metabolite were screened, allowing us to investigate structure–activity relationships (SAR). Several synthetic analogs exhibited an effect on the Ca_V_3.1 channel. The purpurealidin analogs **74**, **76**, **79,** and **99** were found to potently inhibit Ca_V_3.1 current, and their activity on Ca_V_3.1-expressing oocytes was electrophysiologically characterized.

## 2. Results

### 2.1. Structure–Activity Relationship of Sponge-Derived Compounds

**Figure 1:**
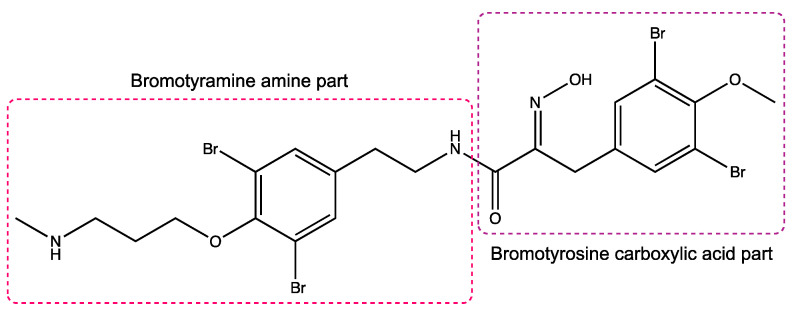
Structure of purpurealidin I (compound **1**) [22].

**Figure 2:**
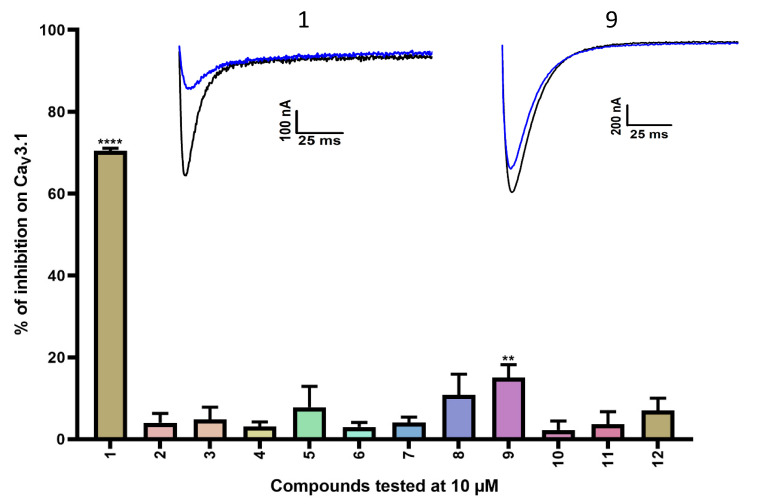
Inhibition of Ca_V_3.1 after application of purpurealidin analogs (compounds **1**–**12**). Compounds were tested at 10 μM. Data are presented as means ± SEM (*n* ≥ 3). Statistical significance was determined using one-way ANOVA with Dunnett’s post-test; ** *p* < 0.01; **** *p* < 0.0001. The inset shows representative Ca_V_3.1 current traces in control (black) and after application of 10 μM compound **1** or compound **9** (blue).

Secondary metabolites of marine sponges are known to possess a wide array of interesting bioactivities and, therefore, their effect on the cardiac T-type Ca_V_ channel Ca_V_3.1 was evaluated. A first screening of purpurealidin I (1) (Figure 1) showed that this alkaloid was able to potently inhibit the rat Ca_V_3.1 channel (Figure 2). In previous work, a library of 119 simplified analogs was synthesized with the aim of targeting K_V_10.1 [18,23,24,25]. Due to the interesting activity of purpurealidin I (1), we decided to repurpose this existing library to investigate the structure–activity relationship with respect to the Ca_V_3.1 channels. All compounds were first dissolved in DMSO and then diluted in a 10 mM Ba^2+^ solution. Compounds were tested at a final concentration of 10 μM.

A first series of compounds is shown in Table S1. Compounds **2**–**12** are simplified analogs that show modifications to the bromotyramine part of the structure of purpurealidin I (Figure 1 and Table S1). The synthesis of these bromotyrosine analogs is described in more detail by Bhat et al. [24]. The average current inhibition (%) is shown in Figure 2.

A first screening showed potent inhibition of Ca_V_3.1 currents by purpurealidin I (compound **1**, 70.5 ± 0.6%). For the simplified analogs, only compound **9** (15.1 ± 3.1%) showed a low but significant inhibition of the Ca_V_3.1 channels. Purpurealidin I (1) showed the strongest inhibition of Ca_V_3.1 currents at 10 μM, but unfortunately, not enough material was available to determine a concentration–response curve for this compound. 

**Figure 3:**
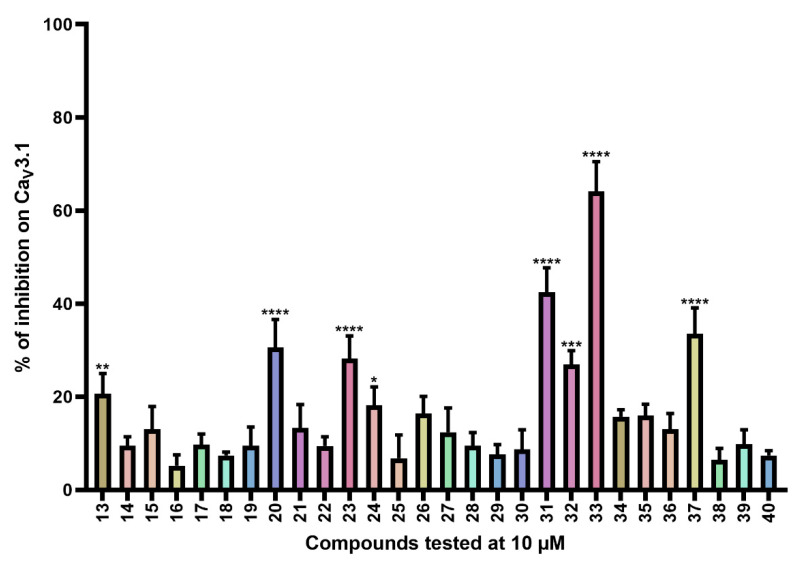
Inhibition of Ca_V_3.1 after application of purpurealidin analogs (compounds **13**–**40**). Compounds were tested at 10 μM. Data are presented as means ± SEM (*n* ≥ 3). Statistical significance was determined using one-way ANOVA with Dunnett’s post-test; * *p* < 0.05; ** *p* < 0.01; *** *p* < 0.001; **** *p* < 0.0001.

Next, compounds **13**–**40** (Table S2) are simplified analogs that show modifications to the bromotyrosine part of the purpurealidin I (1) structure (Figure 1). More specifically, these compounds are simpler amide analogs containing the tyramine fragment in combination with substituted phenyl rings (Ar in Table S2). The synthesis of these bromotyramine analogs is described in more detail by Moreels et al. [18]. The average current inhibition (%) is shown in Figure 3.

Ca_V_3.1 currents were significantly inhibited by compounds **13** (20.7 ± 4.3%), **20** (30.6 ± 6.0%), **23** (28.2 ± 4.9%), **24** (18.2 ± 3.9), **31** (42.4 ± 5.3%), **32** (26.9 ± 3.0%), **33** (64.1 ± 6.4%), and **37** (33.5 ± 5.6%). 

**Figure 4:**
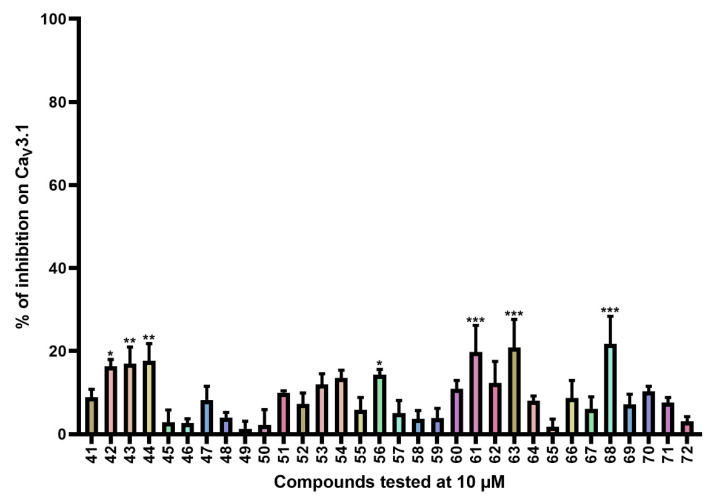
Inhibition of Ca_V_3.1 after application of purpurealidin analogs (compounds **41**–**72**). Compounds were tested at 10 μM. Data are presented as means ± SEM (*n* ≥ 3). Statistical significance was determined using one-way ANOVA with Dunnett’s post-test; * *p* < 0.05; ** *p* < 0.01; *** *p* < 0.001.

Compounds **41**–**72** (Table S3) consist of synthesized marine bromotyrosine clavatadine C **41** and its spiro-structured analogs, which here are called spirocyclic bromotyrosines. Structurally, these analogs are more rigid and occupy the chemical space better than the open-chain bromotyrosine. The synthesis of these compounds is described in more detail by Patel et al. [23]. Synthesis of chloro spiro compounds **65**–**72** is described in the supporting material (Supplemental Scheme S1). The average current inhibition (%) is shown in Figure 4.

Ca_V_3.1 currents were significantly inhibited by compounds **42** (16.3 ± 1.7%), **43** (17.0 ± 4.0%), **44** (17.7 ± 4.1%), **61** (19.8 ± 6.4%), **63** (20.9 ± 6.7%), and **68** (21.7 ± 6.7%).

**Figure 5:**
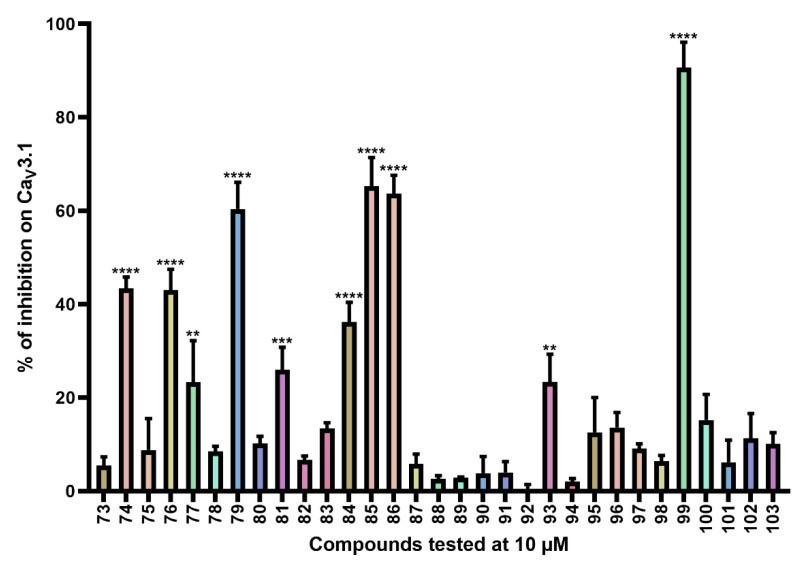
Inhibition of Ca_V_3.1 after application of purpurealidin analogs (compounds **73**–**103**). Compounds were tested at 10 μM. Data are presented as means ± SEM (*n* ≥ 3). Statistical significance was determined using one-way ANOVA with Dunnett’s post-test; ** *p* < 0.01; *** *p* < 0.001; **** *p* < 0.0001.

**Figure 6:**
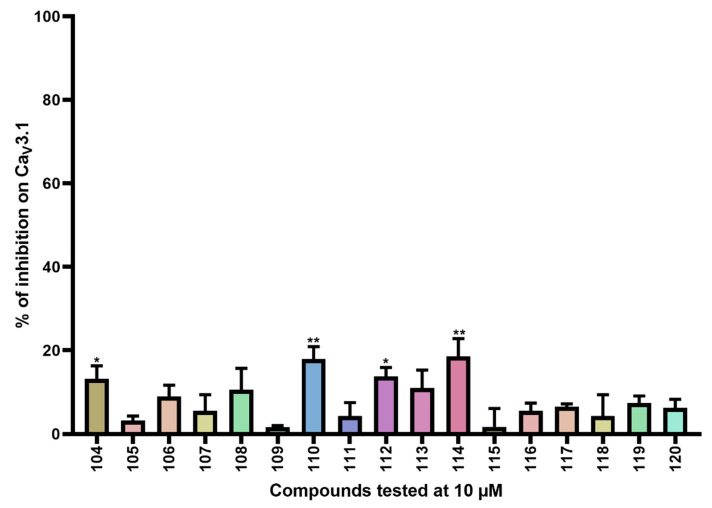
Inhibition of Ca_V_3.1 after application of purpurealidin analogs (compounds **104**–**120**). Compounds were tested at 10 μM. Data are presented as means ± SEM (*n* ≥3). Statistical significance was determined using one-way ANOVA with Dunnett’s post-test; * *p* < 0.05; ** *p* < 0.01.

For the last series of compounds, another strategy has been applied. Based on the structure of the purpurealidin analogs, a pharmacophore model was utilized for the virtual screening of commercially available compounds. One of the hit compounds was compound **76** (Table S4). This compound has a unique diarylamine structure and, therefore, represents a new structural class of Ca_V_3 channel inhibitors. Compounds **73**–**75** and **77**–**119** (Tables S4 and S5) are analogous structures to compound **76**. The synthesis of these compounds is described in more detail by Toplak et al. [25] and Gubič et al. [26]. The average current inhibition (%) is shown in Figure 5 and Figure 6.

Ca_V_3.1 currents were significantly inhibited by compounds **74** (43.4 ± 2.4%), **76** (43.0 ± 4.5%), **77** (23.3 ± 8.9%), **79** (60.4 ± 5.7%), **81** (26.0 ± 4.8%), **84** (36.2 ± 4.2%), **85** (65.3 ± 6.1%), **86** (63.7 ± 3.9%), **93** (23.4 ± 5.9%), and **99** (90.7 ± 5.4%). An electrophysiological characterization is conducted for compounds **74**, **76**, **79,** and **99** and is reported below.

Furthermore, Ca_V_3.1 currents were significantly inhibited by compounds **104** (13.2 ± 3.1%), **110** (17.9 ± 3.0%), **112** (13.8 ± 2.1%), and **114** (18.6 ± 4.2%).

### 2.2. Electrophysiological Characterization of Compounds ***74***, ***76***, ***79***, and ***99*** on the T-Type Ca_V_ Channels

**Figure 7:**
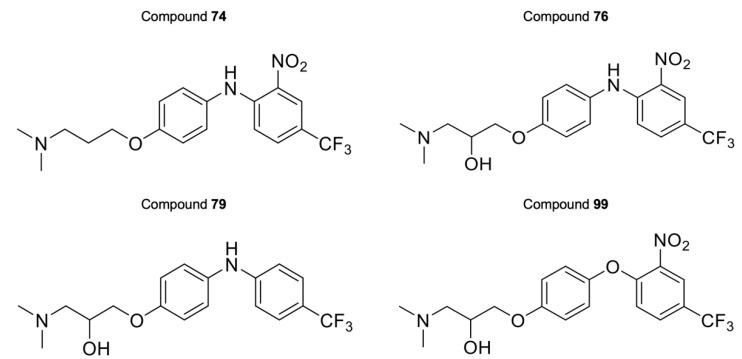
Structures of compounds **74**, **76**, **79**, and **99**.

The activity of the purpurealidin compounds **74**, **76**, **79** and **99** (Figure 7) was investigated using electrophysiology on oocytes that expressed the Ca_V_3.1 channel.

**Figure 8:**
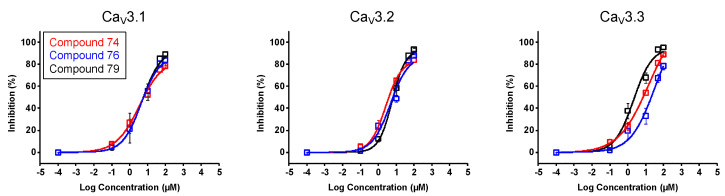
Concentration–response determination for compounds **74**, **76**, and **79**. The concentration dependency was assessed by measuring the current inhibition in the presence of increasing compound concentrations (0.1 μM, 1 μM, 10 μM, 50 μM, and 100 μM). Red curve shows the concentration–response for compound **74**. Blue curve shows the concentration–response for compound **76**. Black curve shows the concentration–response for compound **79**. Data are presented as means ± SEM (*n* ≥ 3).

In a first stage, an electrophysiological characterization was conducted for compounds **74**, **76,** and **79**. The concentration-dependency of the inhibition was evaluated using increasing compound concentrations on all three Ca_V_3 isoforms (Figure 8). The calculated IC_50_ values and Hill coefficients are shown in Table 1. 

**Figure 9:**
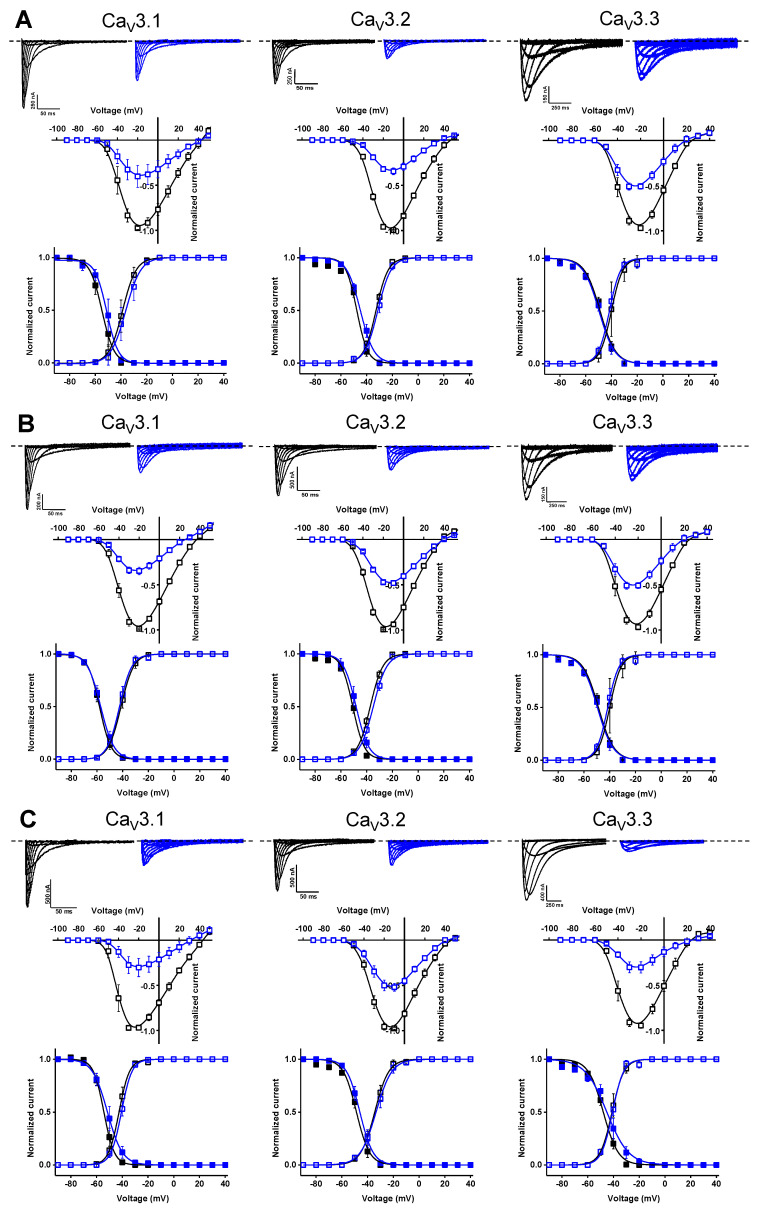
Electrophysiological characterization of compounds **74** (**A**), **76** (**B**), and **79** (**C**) on all three Ca_V_3 channel isoforms. (**A**–**C**) **Top** row: current traces before (black) and after (blue) application of 10 μM compound. In 10 mM Ba^2+^, currents were elicited by depolarizing pulses from −90 to +50 mV for Ca_V_3.1 and Ca_V_3.2, and from −90 to +40 mV for Ca_V_3.3. Holding potential was −90 mV. First 300 ms of the depolarizing pulses are shown for Ca_V_3.1 and Ca_V_3.2, and 600 ms for Ca_V_3.3. **Middle** row: Normalized voltage–current relationship in control (black symbols) and compound (10 μM, blue symbols) conditions. **Bottom** row: steady-state activation (open symbols) and inactivation (closed symbols) curves in control (black symbols) and compound (10 μM, blue symbols) conditions. Data are presented as means ± SEM (*n* ≥ 3).

Further electrophysiological characterization was performed with a compound concentration of 10 μM. Current traces of the Ca_V_3 channel isoforms before (control, black) and after the application of 10 μM compound (blue) are shown in the top row of Figure 9A for compound **74**, the top row of Figure 9B for compound **76**, and the top row of Figure 9C for compound **79**. In the middle row of each panel, the normalized current amplitude (I/I_max,control_) in control conditions (black circles) and compound conditions (blue circles) is plotted against the pulse potential. To evaluate the effect of the compounds on the activation and inactivation of Ca_V_3 channels, the normalized current amplitude (I/I_max_) is plotted against the corresponding pulse potentials and fitted with the Boltzmann equation (Figure 9, bottom row of each panel). No significant shift in the voltage dependency of the Ca_V_3 channels was observed upon compound binding (Figure 9, middle and bottom row of each panel). Wash-in and wash-out studies showed that the binding of these compounds to the Ca_V_3 channels is reversible (Supplemental Figure S1).

In the next stage of the structure–activity relationship studies, we discovered that compound **99** could potently inhibit the Ca_V_3.1 channels. Moreover, for this compound, an electrophysiological characterization was conducted. 

First, the concentration-dependent inhibitory effect of compound **99** was determined by measuring the current inhibition in the presence of increasing compound concentrations. The calculated IC_50_ value and the Hill coefficient of compound **99** on Ca_V_3.1 are 4.1 ± 0.1 μM and 1.6 ± 0.1, respectively. To investigate isoform selectivity between the two cardiac Ca_V_3 channels, the IC_50_ value and Hill coefficient were also determined for Ca_V_3.2. The IC_50_ value is 2.3 ± 0.5 μM with a Hill coefficient of 1.4 ± 0.3. Further electrophysiological characterization was performed with 5 μM of compound **99** on the Ca_V_3.1 channel. 

**Figure 10:**
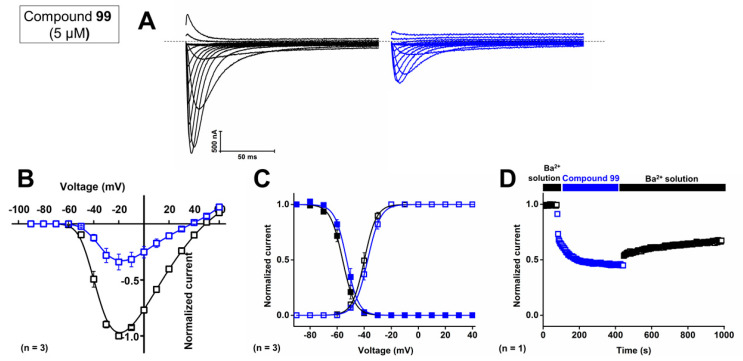
Electrophysiological characterization of activity of compound **99** on the Ca_V_3.1 channel. (**A**) Current traces before (black) and after (blue) application of **5** μM compound **99**. In 10 mM Ba^2+^, currents were elicited by depolarizing pulses from −90 to +60 mV. Holding potential was −90 mV. First 300 ms of the depolarizing pulses are shown. (**B**) Normalized voltage–current relationship in control (black symbols) and compound **99** (5 μM, blue symbols) conditions. (**C**) Steady-state activation (open symbols) and inactivation (closed symbols) curves in control (black symbols) and compound **99** (5 μM, blue symbols) conditions. Data are presented as means ± SEM (*n* = 3). (**D**) Normalized Ca_V_3.1 channel current during wash-in and wash-out of compound **99** over time.

Representative current traces of Ca_V_3.1 are shown in control conditions (black line) and after application of 5 μM compound **99** (blue line, Figure 10A). At a concentration of 5 μM, Ca_V_3.1 currents are inhibited by 57.0 ± 5.8%. The normalized current amplitude (I/I_max,control_) in control conditions (black circles) and compound conditions (blue circles) is plotted against the pulse potential (Figure 10B). Next, the effect of the compounds on the activation and inactivation of the Ca_V_3.1 channels is investigated by plotting the normalized current amplitude (I/I_max_) against the corresponding pulse potentials and by fitting this with the Boltzmann equation. Steady-state activation and inactivation curves showed no significant shift in the voltage dependency of Ca_V_3.1 channels upon compound binding (Figure 10B,C). For the midpoint of activation, V_1/2_ values yielded −39.9 ± 0.1 mV in control conditions and −37.4 ± 0.1 mV in the presence of 5 μM compound **99**. For the inactivation curves, the V_1/2_ shifted from −56.0 ± 0.1 mV to −53.0 ± 0.2 mV in control and compound situations, respectively. In Figure 10D, a representative normalized time-dependent profile of the Ca_V_3.1 currents during wash-in and wash-out studies of 5 μM compound **99** is shown for one experiment. The effect of the compound was not completely reversible upon wash-out with compound-free bath solution (Figure 10D). Unfortunately, due to the limited amount available, compound 99 could not be tested on Ca_V_3.3 channels.

### 2.3. Selectivity Screening

As these purpurealidin analogs represent a new class of potent Ca_V_3.1 inhibitors, they could potentially be used as pharmacological tools to investigate the role of Ca_V_3 channels in various disease models. Therefore, compounds **74**, **76**, **79**, and **99** were further evaluated at a concentration of 10 μM for their selectivity against cardiac voltage-gated potassium and sodium channels. No significant inhibition was observed for compounds **74**, **76**, **79**, and **99** against K_V_1.4, K_V_4.2 and Na_V_1.5 channels. Nevertheless, the affinity for the K_V_11.1 channel is an important issue for all four compounds (Table 2). 

## 3. Discussion

### 3.1. Structure–Activity Relationship

In this study, we report the identification and characterization of a new group of Ca_V_3 channel blockers. These compounds are analogs based on the structure of purpurealidin I (1). Both the bromotyrosine and bromotyramine parts of the structure have been investigated.

In the first series of compounds (Table S1), the bromotyramine part of the purpurealidin I structure was modified. Only compound **9** showed a low but significant inhibition of 15.1 ± 3.1%. An interesting comparison in structure can be made between purpurealidin I (1) and compound **4**. Although their bromotyrosine part is identical, the bromotyramine part of these compounds shows a few differences. The aryl group of compound **1** contains two bromine atoms in the meta position, whereas compound **4** contains only one. Furthermore, compound **4** lacks an ethylmethylamine group that is present in compound **1**. Finally, the linker between hydroxyamino amide and the aryl group of compound **1** contains two carbon atoms, whereas this linker is missing in compound **4**. These modifications cause a difference in inhibition of approximately 70%. When the structure of compound **4** is compared to the structure of compound **9**, only a small difference in structure can be noticed. Compound **4** is halogenated with a bromine atom, whereas compound **9** is halogenated with a chlorine atom, causing a shift in effect from not significant (compound **4**) to significant (compound **9**) inhibition of the Ca_V_3.1 currents.

In the next series of compounds (Table S2), structural changes have been made to the bromotyrosine part of purpurealidin I (1). The three most potent compounds from this series are compounds **31**, **33**, and **37,** as they produce 42.4 ± 5.3%, 64.1 ± 6.4%, and 33.5 ± 5.6% inhibition, respectively. Remarkably, all three analogs show a minor difference in the structure that sets them apart from the other compounds in this series. They have a monomethylamine at the end of the propoxy chain, which is identical to the purpurealidin I (1) structure, whereas this is replaced by an *N*,*N*-dimethylamino moiety in the other compounds, except for compound **26**. In compounds **16** and **19**, the monomethylamine is replaced by isopropyl, and no significant effect could be observed. Nonetheless, it is uncertain whether the loss of activity can be attributed to this or to the *para*-methoxy group that is missing in the aryl group (Ar). Between the three analogs **31**, **33**, and **37**, the only difference is the halogenation of the *meta*-position, which influences the activity as follows: dihalogenation with chlorine atoms (compound **33**) > monohalogenation with a chlorine atom (compound **31**) > dihalogenation with fluorine atoms (compound **37**). It appears that compounds dihalogenated with chlorine atoms show a greater effect. A similar effect is seen when two other compounds are compared: compound **30** is monohalogenated with a chlorine atom and caused 8.7 ± 4.2% inhibition; compound **32** is dihalogenated with chlorine atoms and inhibited the channels with 26.9 ± 3.0%. Although the tyrosine part of compound **1** is halogenated with two bromine atoms, halogenation with bromine or iodine atoms appears to cause a loss of activity for this series of analogs. Further investigation is required to understand the structurally important moieties in detail.

The third series of compounds (Table S3) include the spirocyclic bromotyrosines. For these analogs, the open chain of the bromotyrosine part is rendered spirocyclic, and the bromotyramine part is replaced by different side chains. Although seven analogs retained some significant activity, it appears that the spirocyclic structure causes a decrease in activity. This difference could be attributed to the more rigid structure of the spirocyclic bromotyrosine analogs. Because there are no large differences in activity between the analogs of this series, it is difficult to determinate any SAR. One thing we could confirm is the importance of halogenation. Non-halogenated compounds showed no significant effect. For the halogenated compounds, no significant difference was observed between bromine or chlorine dihalogenation for compounds with identical R-groups, for example, compounds **48** and **65**, compounds **53** and **68**, compounds **55** and **66**, and compounds **56** and **67**. However, a significant difference was observed between the activity of compound **68** (21.7 ± 6.7% inhibition) and the activity of compounds **58** (3.7 ± 2.0% inhibition) and **72** (3.1 ± 1.1% inhibition). Remarkably, there is only a minor structural difference. All three compounds contain the same pyridine ring, but the pyridine ring of compound **68** is immediately linked to the amide. However, compounds **58** and **72** contain a methylene unit between the pyridine ring and the amide, which completely abolishes the effect.

Finally, the last series comprises the diarylamine analogs (Table S4). Compound **99** is the most active compound of this series, with an inhibition of 90.7 ± 5.4%. This analog has a unique structure compared to other analogs of this series. Although the diarylamine group is a common feature among most compounds, it is replaced by a diarylether group in compound **99**. Other features of this compound include an *ortho*-nitro and *para*-trifluoromethyl substituent on the phenyl ring (R1 and R2 in Table S4). Furthermore, at the end of the aliphatic chain is a basic dimethylamine group (R3 in Table S4), and finally, the aliphatic chain between the aromatic ring and the basic center contains a hydroxy group (R4 in Table S4). An interesting comparison can be made between compounds **99** and **76**. The latter compound shares the same features as compound **99**, with the only exception that it contains the diarylamine structure instead of the diarylether group. This structural difference reduced the activity of compound **76** by half compared to compound **99**. Another noteworthy comparison can be made between compounds **76** and **79**. Removal of the nitro group on the phenyl ring of compound **76** causes an increase in activity by approximately 20% for compound **79**, although this difference was not shown to be significant. When the nitro group on the phenyl ring is replaced by a methyl ester (compound **81**), no difference in activity could be observed. However, compound **77**, in which the nitro group is replaced by an amino group, lost approximately 20% activity compared to compound **76**. For two analogs, compounds **73** and **74**, the hydroxy group was removed. Despite this structural modification, no difference in effect was observed between these compounds and their analogs that contain the hydroxy group (compounds **75** and **76**, respectively). Furthermore, a significant increase in activity was observed for compounds that contain a *para*-trifluoromethyl group versus the ones without, for example, compounds **73** and **74**, **75** and **76**, **78** and **79**, and **80** and **81**. Replacement of the amine linker between the two aryl groups by an amide, resulting in an *N*-aryl-arylamide structure (such as compounds **102** and **103**), abolished all activity. Finally, it appears that compounds with a large group at the end of the aliphatic chain are less potent. This is observed in compounds with a morpholino moiety (such as compounds **88**–**93**) or an aniline moiety (compound **94**), or even in larger groups such as compounds **95**–**98**. Remarkably, a structural difference was noticed that is similar to what is earlier described for the bromotyramine analogs (second series, Table S2). Namely, at the end of the aliphatic chain, compound **85** contains a monomethylamine that is identical to the purpurealidin I (1) structure, causing a higher percentage of inhibition than its analogous compound with an *N*,*N*-dimethylamino moiety (compound **76**).

The other diarylamine analogs (Table S5) show no big changes in effect, although a few differences may be worth mentioning. First, no significant difference in activity was observed upon non-halogenation, bromine dehalogenation, or chlorine dihalogenation of the aryl ring. Next, compound **104** shows a structure that is comparable with compound **74**. They both contain an *ortho*-nitro and *para*-trifluoromethyl substituent on the phenyl ring, and they do not show a hydroxy group on their aliphatic chain. The only difference is that the aliphatic chain of compound **104** lacks one carbon atom compared to compound **74**. This causes a reduction in activation for compound **104** by approximately 30%. Lastly, compounds **115**–**119** have an *N*-aryl-arylamide structure, contain a phenyl ring directly linked to the ether that connects it to the aryl ring, and show alterations in the positioning of the nitro groups. Those differences in structure caused a complete loss of activity. However, further structure–function studies are required to draw more definite conclusions. 

### 3.2. Electrophysiological Characterization

After observing the interesting effect of compounds **74**, **76**, **79**, and **99** on Ca_V_3.1 channels, further characterization was conducted. The calculated IC_50_ values yielded 2.6 ± 0.6 μM, 3.7 ± 0.6 μM, 5.9 ± 1.9 μM, and 4.1 ± 0.1 μM, respectively. No significant difference between these values was observed, although, at a concentration of 10 μM, the effect of compound **99** was significantly higher. These variances may be explained by differences in the properties of the compounds. Namely, the effect of compounds **74**, **76**, and **79** on all three Ca_V_3 channels were shown to be reversible, but this seemed not the case for compound **99**. After up to ten minutes of washing out with a compound-free solution, the effect of the compound was still present. Furthermore, our electrophysiological studies suggested that these compounds act like a pore blocker obstructing the ion flow by binding in the pore region of the Ca_V_3.1 channel rather than acting as a voltage-sensor modifier because no significant shift in the activation and steady-state inactivation curves could be observed.

**Figure 11:**
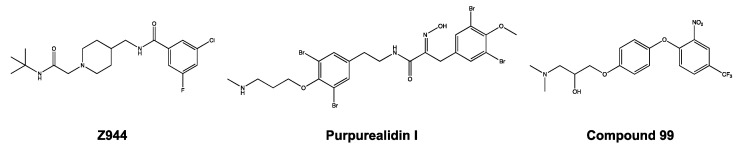
Structures of Z944, purpurealidin I (1), and compound **99**.

Zhao et al. observed a similar phenomenon, in that the Z944 compound acted as a pore blocker on the Ca_V_3.1 channel [27]. After detailed investigations, they showed that the Z944 compound exhibited an arch-shaped conformation, reclining in the central cavity of the pore domain, with its wide end embedded in the II–III fenestration and its narrow end situated above the intracellular gate like a plug [27]. It may be noticed that the structure of purpurealidin I (1) and compound **99** resembles the Z944 structure (Figure 11). This could indicate that our compounds share the same binding location, which would be consistent with our findings that the compounds are pore blockers of the Ca_V_3.1 channel.

### 3.3. Selectivity Screening

In the last part of this study, analogs **74**, **76**, **79**, and **99** were evaluated for their selectivity against other cardiac ion channels, in particular the K_V_11.1 channel. The currents of potassium channels, K_V_1.4 and K_V_4.2, and the sodium channel, Na_V_1.5, play an important role in the generation of action potentials in the heart [28]. Therefore, the effect of the compounds was investigated on these channels, but no activity could be observed. What is of concern, however, is their activity on K_V_11.1 channels. All four compounds were shown to potently inhibit hERG channels.

Whereas compound **99** is equipotent across Ca_V_3.1 and hERG channels, Z944 is 150-fold more potent against Ca_V_3.1 compared to hERG channels. Remarkably, the hERG activity of compound **99** (69.1 ± 1.9% at a concentration of 10 μM) is comparable to the hERG activity of Z944 (IC_50_ = 7.8 μM).

This selectivity issue is of major concern during drug development projects. Inhibition of the hERG channel can cause prolongation of the AP and lead to an increase in the length of time between the start of the Q-wave and the end of the T-wave on an electrocardiogram (QT interval), resulting in lethal cardiac arrhythmia, e.g., Torsade de Pointes (TdP) [29]. Jamieson et al. summarized different in vitro, in vivo, and in silico approaches to determine and overcome hERG blockade. They suggest disrupting any putative π-stacking interactions with the channel, which, in the case of compound **99**, could be related to its diarylether group. Furthermore, they propose attenuating hERG inhibition by creating subtle changes in the molecular architecture, such as by removing electron-donating groups, adding electron-withdrawing moieties, or modifying the template of the structure, such as by deleting distal aromatic groups or incorporating heterocyclic groups [29]. These techniques can be used in the future to further optimize our lead compounds. Moreover, the published cryo-EM structure of the hERG channel can help in predicting the hERG liability of compounds [30].

## 4. Materials and Methods

### 4.1. Compound Synthesis

For this study, we made use of an existing library of purpurealidin analogs. These compounds were chemically synthesized in previous studies, as described in detail by Moreels et al. [18], Patel et al. [23], Bhat et al. [24], Toplak et al. [25], and Gubič et al. [26]. Synthesis of compounds **65**–**72** and **115**–**120** is described in the supporting material.

### 4.2. Xenopus Laevis Surgery

Stage V–VI oocytes were isolated via partial ovariectomy from *X. laevis* frogs (African clawed frogs), as described previously [31]. Mature female frogs were purchased from CRB Xénopes (Rennes, France) and housed in the Aquatic Facility (KU Leuven) in compliance with the regulations of the European Union (EU) concerning the welfare of laboratory animals, as declared in Directive 2010/63/EU. After the frogs were anesthetized by a 15 min submersion in 0.1% tricaine methanesulfonate (pH 7.0), the oocytes were collected. The isolated oocytes were then washed with a 1.5 mg/mL collagenase solution to remove the follicle layer.

### 4.3. Expression of Ca_V_3 Channels in Xenopus Laevis Oocytes

Rat Ca_V_3.1, human Ca_V_3.2, rat Ca_V_3.3, human Na_V_1.5, rat K_V_1.4, rat K_V_4.2, and human K_V_11.1 were expressed in *X. laevis* oocytes by linearizing the plasmids and subsequent in vitro transcription using a commercial T7 mMESSAGE mMACHINE transcription kit (Ambion, Carlsbad, CA, USA). Defolliculated *Xenopus* oocytes were injected with 50 nL of cRNA at a concentration of 1 ng/nL by using a microinjector (Drummond Scientific Company, Broomall, PA, USA). The oocytes were incubated in a solution containing (in mM): NaCl, 96; KCl, 2; CaCl_2_, 1.8; MgCl_2_, 2; and HEPES, 5 (pH 7.5). This solution was supplemented with 50 mg/L gentamicin sulfate and 90 mg theophylline.

### 4.4. Electrophysiological Recordings

Two-electrode voltage clamp recordings were performed at room temperature (18–22 °C) by using a GeneClamp 500 amplifier (Molecular Devices, San Jose, CA, USA) controlled by a pClamp data acquisition system (Axon Instruments, Union City, CA, USA). Whole-cell currents from oocytes were recorded 5–10 days after mRNA injection. The bath solution composition was the following (in mM): BaCl_2_, 10; NaOH, 90; KOH, 1; EDTA, 0.1; and HEPES, 5 (pH 7.5). Voltage and current electrodes were filled with 3 M KCl. Resistances of both electrodes were kept between 0.8 and 1.5 MΩ. The elicited Ca_V_3.1, Ca_V_3.2, and Ca_V_3.3 currents were filtered at 2 kHz and sampled at 4 kHz using a four-pole low-pass Bessel filter. Leak subtraction was performed by using a -P/4 protocol. Oocytes were placed in a measuring chamber filled with 200 μL ND96. The compounds were added directly to the measuring chamber under continuous application of the described voltage protocol. The percentage channel modulation was calculated when steady-state conditions were reached.

For the electrophysiological analysis of peptides, a number of protocols were applied from a holding potential of −90 mV. Currents for Ca_V_3.1 and Ca_V_3.2, which were carried by Ba^2+^, were evoked by 300 ms depolarizing pulses to −25 mV or Vmax (the voltage corresponding to maximal Ba^2+^ current in control conditions). Currents for Ca_V_3.3, which were carried by Ba^2+^, were evoked by 1 s depolarizing pulses to −25 mV or Vmax. The current–voltage relationships were determined by 600 ms step depolarizations between −90 and +60 mV, using 10 mV increments. The values of *I_P_*_Ba_ were normalized to the maximal Ba^2+^ current amplitude and plotted as a function of voltage. To investigate the effect of the peptide toxins on the activation and steady-state inactivation, a standard 2-step protocol was used. In this protocol, 600 ms conditioning 10 mV step prepulses, ranging from −90 to +60 mV, were followed by a 400 ms test pulse to −25 mV. Data were normalized to the maximal Ba^2+^ current amplitude, plotted against prepulse potential, and fitted by using the Boltzmann function: *I_Ca_*/*I_max_* = {(1 − C)/(1 + exp[(*V* − *V_h_*)/*k*)]} + C, where *I_max_* is the maximal *I_Ba_*, *V_h_* is the voltage corresponding to half-maximal inactivation, *V* is the test voltage, *k* is the slope factor, and C is a constant representing a non-inactivating persistent fraction (close to 0 in control). The concentration–response relationship was determined by fitting the data with the Hill equation: *y* = 100/{1 + [IC_50_/(toxin)]*^h^*}, where *y* is the amplitude of the toxin-induced effect, IC_50_ is the toxin concentration at half-maximal efficacy, toxin is the toxin concentration, and *h* is the Hill coefficient.

The selectivity screening was conducted with the following protocols: the elicited K_V_1.4, K_V_4.2, and K_V_11.1 currents were filtered at 0.5 kHz and sampled at 2 kHz; Na_V_1.5 currents were filtered at 2 kHz and sampled at 20 kHz using a four-pole low-pass Bessel filter. Leak subtraction was performed using a -P/4 protocol. The oocytes were measured in a bath solution composition of ND96 (in mM): NaCl, 96; KCl, 2; CaCl_2_, 1.8; MgCl_2_, 2; and HEPES, 5 (pH 7.5). For the electrophysiological analysis of the compounds, a number of protocols were applied from a holding potential of −90 mV. Currents for K_V_1.4 and K_V_4.2 were evoked by 0.5 s depolarizing pulses to 0 mV, followed by 0.5 s repolarizing pulses to −50 mV. Currents for K_V_11.1 were evoked by 2.5 s depolarizing prepulses to +40 mV, followed by hyperpolarizing pulses to −120 mV for 2.5 s. Currents for Na_V_1.5 were evoked by 100 ms depolarizing pulses to 0 mV.

### 4.5. Data Analysis

All electrophysiological data are presented as means ± SEM of at least three independent experiments (*n* ≥ 3), unless otherwise indicated. All data were analyzed using pClamp Clampfit 10.4 (Molecular Devices, Downingtown, PA, USA) and OriginPro 9 (Originlab, Northampton, MA, USA) or GraphPad Prism 8 software (GraphPad Software, Inc., San Diego, CA, USA). Statistical significance was determined using one-way ANOVA with Dunnett’s post-test or one-way ANOVA with Tukey’s post-test; * *p* < 0.05; ** *p* < 0.01; *** *p* < 0.001; **** *p* < 0.0001.

## 5. Conclusions

In this study, we discovered a new class of Ca_V_3 inhibitors. The effect of the sponge-derived purpurealidin I (1) compound and 119 bromotyrosine analogs were examined using two-electrode voltage clamp electrophysiology on oocytes expressing the Ca_V_3.1 channel. Although some conclusions could be drawn, additional studies are still required to further analyze and correlate differences in structure with differences in activity on the Ca_V_3.1 channel. Moreover, molecular modeling can be used to determine the binding location of these compounds in the Ca_V_3.1 channel. Further electrophysiological characterization was conducted with potent inhibitor compounds **74**, **76**, **79**, and **99**. Our data suggest that these compounds act on the channel as pore blockers.

## Figures and Tables

**Table 1:** IC_50_ values and Hill coefficients obtained for compounds **74**, **76,** and **79**, on Ca_V_3 channel isoforms.

	Compound 74		Compound 76		Compound 79	
	IC_50_	Hill Coefficient	IC_50_	Hill Coefficient	IC_50_	Hill Coefficient
**Ca_V_3.1**	2.6 ± 0.6	0.8 ± 0.1	3.7 ± 0.6	0.9 ± 0.1	5.9 ± 1.9	0.8 ± 0.1
**Ca_V_3.2**	3.0 ± 0.2	0.8 ± 0.0	8.4 ± 3.6	0.8 ± 0.1	7.0 ± 0.1	1.0 ± 0.0
**Ca_V_3.3**	13.9 ± 5.1	0.5 ± 0.1	18.2 ± 3.1	0.7 ± 0.1	2.7 ± 0.3	0.7 ± 0.1

**Table 2:** Percentages of inhibition of the selectivity screening of the selected compounds against cardiac voltage-gated potassium and sodium channels at a concentration of 10 μM. Data are presented as means ± SEM (*n* ≥ 3). NS—not significant.

	Compound 74	Compound 76	Compound 79	Compound 99
**K_V_11.1**	45.8 ± 2.9%	64.2 ± 3.2%	67.6 ± 3.9%	69.1 ± 1.9%
**K_V_1.4**	NS	NS	NS	NS
**K_V_4.2**	NS	NS	NS	NS
**Na_V_1.5**	NS	NS	NS	NS

## Data Availability

Not applicable.

## Supplementary Materials

The following supporting information can be downloaded at: https://www.mdpi.com/article/10.3390/ijms24043429/s1.
