# Supplementary material for: Structure-Function Studies of Sponge-Derived Compounds on the Cardiac CaV3.1 Channel
Source: Int J Mol Sci. 2023 Feb 8;24(4):3429. doi: 10.3390/ijms24043429 (PMC9962600; doi:10.3390/ijms24043429)

Table S1. Structures of purpurealidin analogs (compounds **1–12**).

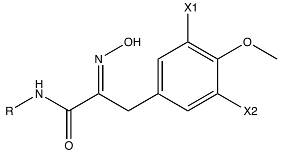

#	X1	X2	R	#	X1	X2	R
**1**	Br	Br	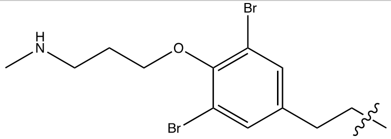	**7**	Br	Br	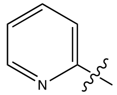
**2**	Br	Br	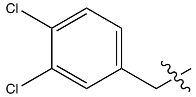	**8**	Br	Br	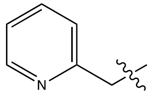
**3**	Br	Br	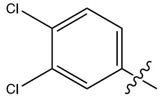	**9**	Br	Br	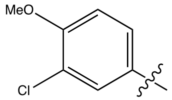
**4**	Br	Br	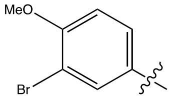	**10**	H	H	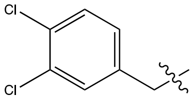
**5**	Br	Br	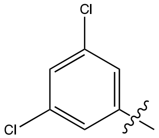	**11**	Br	H	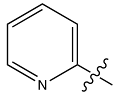
**6**	Br	Br	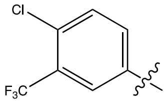	**12**	Br	H	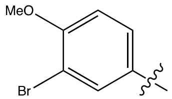

Table S2. Structures of purpurealidin analogs (compounds **13**–**40**).

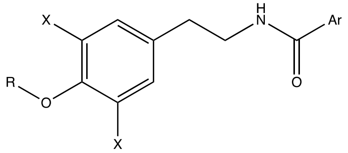

#	Ar	X	R	#	Ar	X	R
**13**	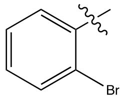	Br	$(CH_2)_3$-$N(CH_3)_2$	**27**	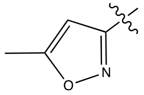	Br	$(CH_2)_3$-$N(CH_3)_2$
**14**	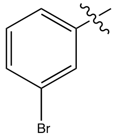	Br	$(CH_2)_3$-$N(CH_3)_2$	**28**	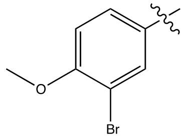	Br	$(CH_2)_3$-$N(CH_3)_2$
**15**	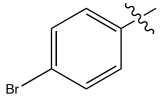	Br	$(CH_2)_3$-$N(CH_3)_2$	**29**	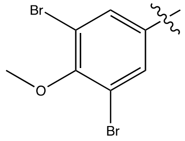	Br	$(CH_2)_3$-$N(CH_3)_2$
**16**	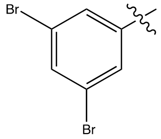	Br	$(CH_2)_3$-CH-$(CH_3)_2$	**30**	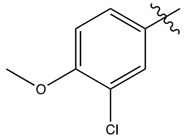	Br	$(CH_2)_3$-$N(CH_3)_2$
**17**	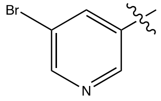	Br	$(CH_2)_3$-$N(CH_3)_2$	**31**	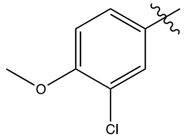	Br	$(CH_2)_3$-NH-CH_3
**18**	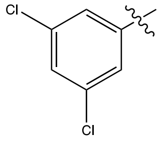	Br	$(CH_2)_3$-$N(CH_3)_2$	**32**	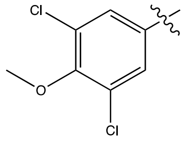	Br	$(CH_2)_3$-$N(CH_3)_2$

**19**	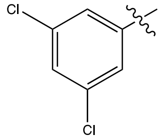	Br	$(CH_2)_3$-CH-$(CH_3)_2$	**33**	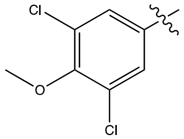	Br	$(CH_2)_3$-NH-CH_3
**20**	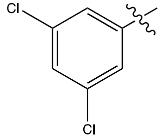	H	$(CH_2)_3$-$N(CH_3)_2$	**34**	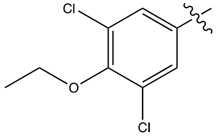	Br	$(CH_2)_3$-$N(CH_3)_2$
**21**	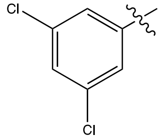	H	H	**35**	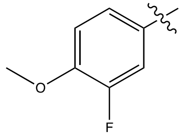	Br	$(CH_2)_3$-$N(CH_3)_2$
**22**	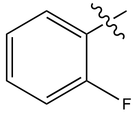	Br	$(CH_2)_3$-$N(CH_3)_2$	**36**	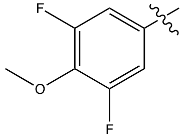	Br	$(CH_2)_3$-$N(CH_3)_2$
**23**	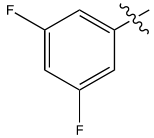	Br	$(CH_2)_3$-$N(CH_3)_2$	**37**	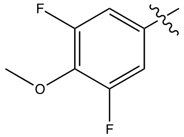	Br	$(CH_2)_3$-NH-CH_3
**24**	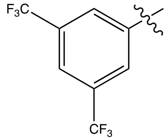	Br	$(CH_2)_3$-$N(CH_3)_2$	**38**	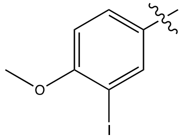	Br	$(CH_2)_3$-$N(CH_3)_2$
**25**	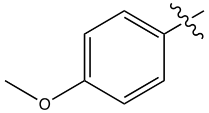	Br	$(CH_2)_3$-$N(CH_3)_2$	**39**	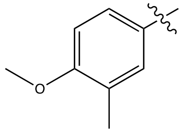	Br	$(CH_2)_3$-$N(CH_3)_2$
**26**	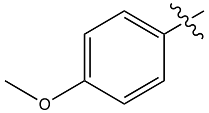	Br	$(CH_2)_3$-NH-CH_3	**40**	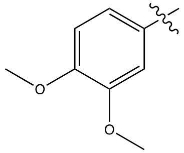	Br	$(CH_2)_3$-$N(CH_3)_2$

Table S3. Structures of purpurealidin analogs (compounds **41–72**).

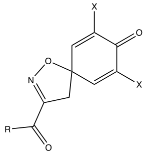

#	X	R	#	X	R
**41**	H	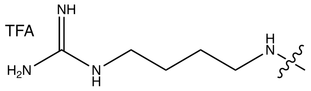	**57**	H	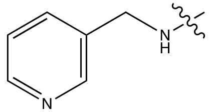
**42**	Br	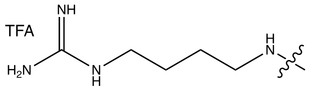	**58**	Br	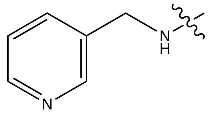
**43**	Br	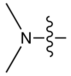	**59**	H	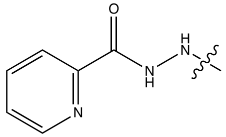
**44**	Br	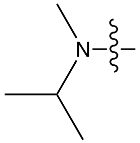	**60**	Br	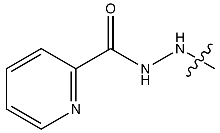
**45**	H	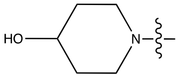	**61**	Br	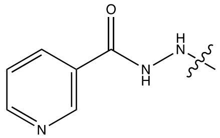
**46**	H	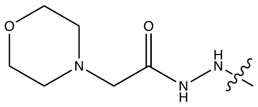	**62**	Br	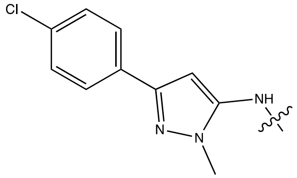

**47**	Br	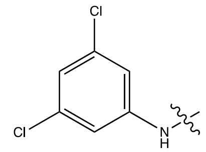
**48**	Br	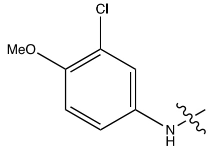
**49**	H	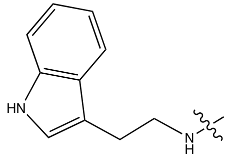
**50**	H	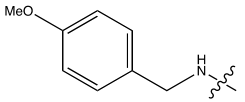
**51**	Br	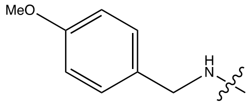
**52**	H	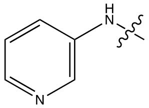
**53**	Br	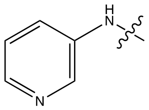
**54**	Br	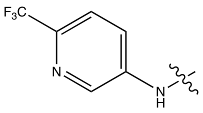
**55**	Br	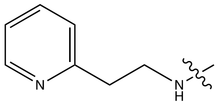
**63**	Br	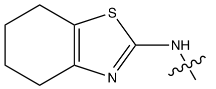
**64**	H	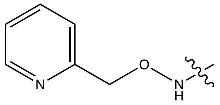
**65**	Cl	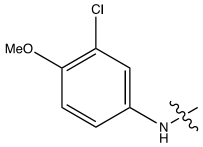
**66**	Cl	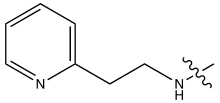
**67**	Cl	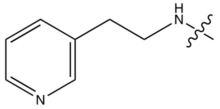
**68**	Cl	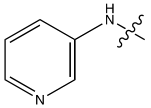
**69**	Cl	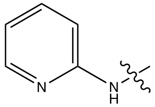
**70**	Cl	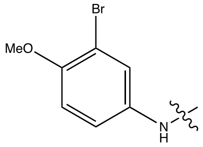
**71**	Cl	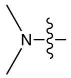

**56**	Br	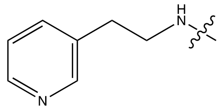	**72**	Cl	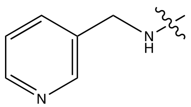

Table S4. Structures of purpurealidin analogs (compounds **73–103**).

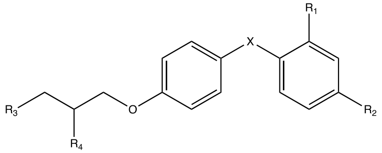

#	X	R1	R2	R3	R4	#	X	R1	R2	R3	R4
**73**	NH	NO_2	H	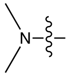	H	**89**	NH	NO_2	CF_3	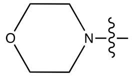	OH
**74**	NH	NO_2	CF_3	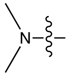	H	**90**	NH	COOMe	H	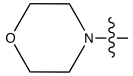	OH
**75**	NH	NO_2	H	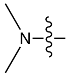	OH	**91**	NH	COOMe	CF_3	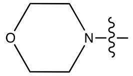	OH
**76**	NH	NO_2	CF_3	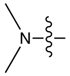	OH	**92**	NH	COOH	H	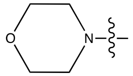	OH
**77**	NH	NH_2	CF_3	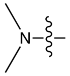	OH	**93**	NH	NO_2	NO_2	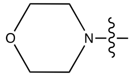	OH
**78**	NH	H	H	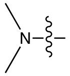	OH	**94**	NH	NO_2	CF_3	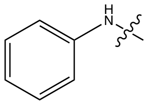	OH
**79**	NH	H	CF_3	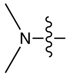	OH	**95**	NH	NO_2	CF_3	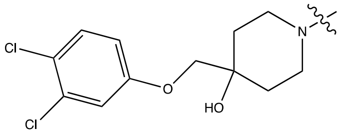	OH

**80**	NH	COOMe	H	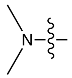	OH	**96**	NH	NO_2	CF_3	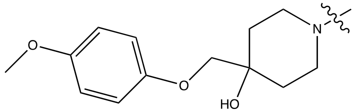	OH
**81**	NH	COOMe	CF_3	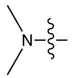	OH	**97**	NH	NO_2	CF_3	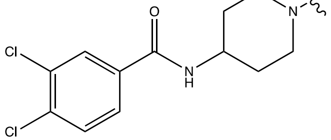	OH
**82**	NH	COOH	H	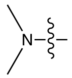	OH	**98**	NH	NO_2	CF_3	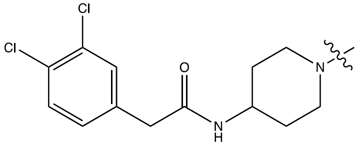	OH
**83**	NH	COOH	CF_3	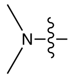	OH	**99**	O	NO_2	CF_3	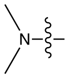	OH
**84**	NH	NO_2	NO_2	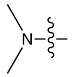	OH	**100**	O	NO_2	CF_3	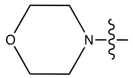	OH
**85**	NH	NO_2	CF_3	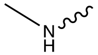	OH	**101**	O	COOMe	H	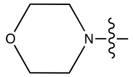	OH
**86**	NH	NO_2	CF_3	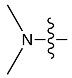	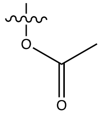	**102**	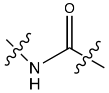	NO_2	CF_3	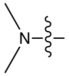	OH
**87**	NH	NO_2	CF_3	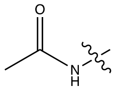	OH	**103**	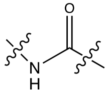	NO_2	CF_3	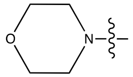	OH
**88**	NH	NO_2	H	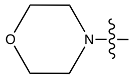	OH						

Table S5. Structures of purpurealidin analogs (compounds **104–120**).

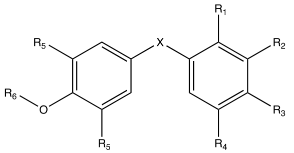

#	X	R1	R2	R3	R4	R5	R6	#	X	R1	R2	R3	R4	R5	R6
**104**	NH	NO_2	H	CF_3	H	H	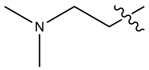	**113**	NH	NO_2	H	CF_3	H	H	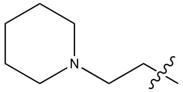
**105**	NH	NO_2	H	CF_3	H	Br	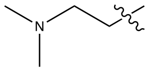	**114**	NH	NO_2	H	CF_3	H	H	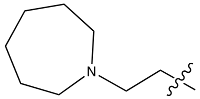
**106**	NH	NO_2	H	CF_3	H	Cl	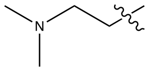	**115**	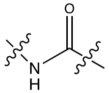	NO_2	H	H	H	H	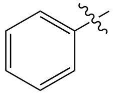
**107**	NH	NO_2	H	CF_3	H	H	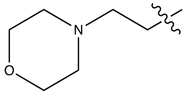	**116**	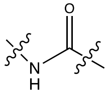	H	NO_2	H	H	H	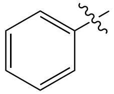
**108**	NH	NO_2	H	CF_3	H	H	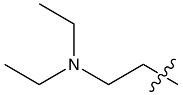	**117**	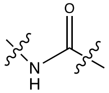	NO_2	H	NO_2	H	H	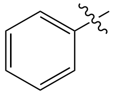
**109**	NH	NO_2	H	CF_3	H	Br	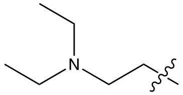	**118**	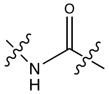	H	NO_2	NO_2	H	H	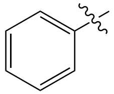
**110**	NH	NO_2	H	CF_3	H	Cl	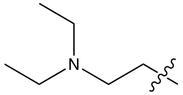	**119**	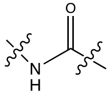	H	NO_2	H	NO_2	H	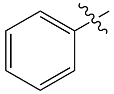
**111**	NH	NO_2	H	CF_3	H	H	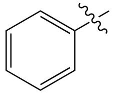	**120**	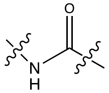	H	NO_2	Cl	H	H	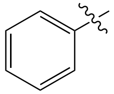

**112**	NH	NO_2	H	CF_3	H	H	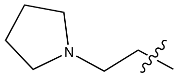

Chemistry: Compounds 65-72

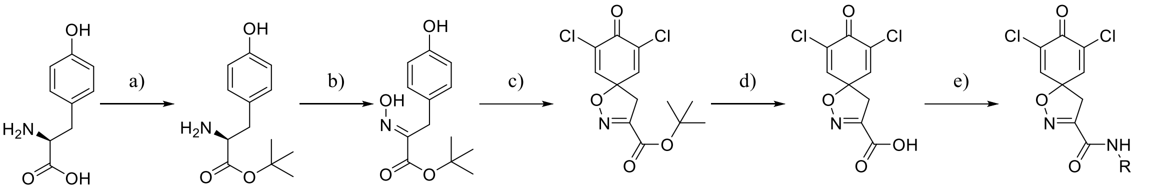

L-tyrosine S1 S2 S3 S4 65-72

Scheme S1. General scheme for synthesis of chlorinated spirocycles **65–72**. Reagents and conditions: a) *tert*-butyl acetate, 20–25 °C, 18 h; b) $Na_2WO_4 \cdot 2H_2O$, H_2O_2, EtOH, 20–25 °C, 7 h; c) NCS, DMF, 20–25 °C, 4 h; d) TFA, DCM, 20–25 °C, 6 h; e) amine, EDC·HCl, HOBt, DCM, 20–25 °C, 5–15 h. The R substituents are given in Table 3.

Synthesis of S1 and **S2** as previously described in Patel, P.A., *et al.* [23]

General

All reactions were carried out using commercially available starting materials unless otherwise stated. ^{1}H NMR and ^{13}C NMR spectra in $CDCl_3$, d_6-acetone or CD_3CN at ambient temperature were recorded on a Bruker Ascend 400 spectrometer. Chemical shifts (δ) are given in parts per million (ppm) relative to the NMR reference solvent signals ($CDCl_3$: 7.26 ppm, 77.16 ppm; d_6-acetone: 2.05 ppm, 29.84 ppm, CD_3CN: 1.94 ppm, 118.26 ppm). Multiplicities are indicated by s (singlet), br s (broad singlet), d (doublet), dd (doublet of doublets), ddd (doublet of doublet of doublets), t (triplet), dt (doublet of triplets), q (quartet), and m (multiplet). The coupling constants *J* are quoted in hertz (Hz). LC-MS and HRMS-spectra were recorded using Waters Acquity UPLC®-system including PDA (with Acquity UPLC® BEH C18 column, 1.7 µm, 50 mm × 2.1 mm, Waters) with Waters Synapt G2 HDMS with the ESI (+), high resolution mode. The mobile phase consisted of H_2O (A) and acetonitrile (B) both containing 0.1% HCOOH. Microwave syntheses were performed in sealed tubes using Biotage Initiator+ instrument equipped with an external IR sensor. The flash chromatography was performed with Biotage Isolera One flash chromatography purification system with 200-800 nm UV-VIS detector using SNAP KP-Sil 10 g, or 25 g cartridges. The TLC plates were provided by Merck (Silica gel 60-F254) and visualization of the amine compounds was done using ninhydrin (a 0.2% w/v solution in a 3% solution of acetic acid in 1-butanol) staining.

***tert*-Butyl 7,9-dichloro-8-oxo-1-oxa-2-azaspiro[4.5]deca-2,6,9-triene-3-carboxylate (S3)**

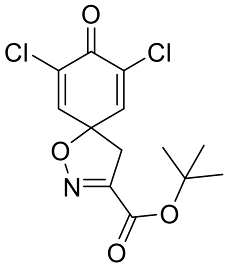

To a solution of *tert*-butyl (*E*)-2-(hydroxyimino)-3-(4-hydroxyphenyl)propanoate P**8** (4.0 g, 0.016 mol) in anhydrous DMF (10 mL), *N*-chlorosuccinimide (7.0 g, 0.053 mol, 3.3 equiv) in anhydrous DMF (27 mL) was added dropwise (15 min). The reaction mixture was stirred at room temperature for 4 h. The mixture was diluted with Et_2O (60 mL), and washed with H_2O (3 × 30 mL) and a 10% solution of $Na_2S_2O_3$ in H_2O (2 × 40 mL). The aqueous phase was back-extracted with Et_2O (4 × 120 mL). The combined organic phases were washed with brine (80 mL), dried over anhydrous Na_2SO_4, filtered, and concentrated *in vacuo*. The crude product was purified with automated flash chromatography (*n*-heptane/EtOAc

gradient: 5→20%) to give the compound S1 as a yellow solid (2.34 g, 46%). ^{1}H NMR (400 MHz, $CDCl_3$) δ 7.05 (s, 2H), 3.43 (s, 2H), 1.57 (s, 9H).

7,9-Dichloro-8-oxo-1-oxa-2-azaspiro[4.5]deca-2,6,9-triene-3-carboxylic acid (S4)

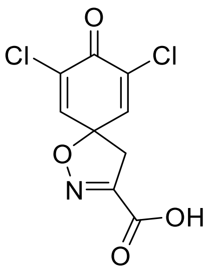

To a solution of *tert*-butyl 7,9-dichloro-8-oxo-1-oxa-2-azaspiro[4.5]deca-2,6,9-triene-3-carboxylate **S1** (0.074 g, 0.2 mmol) in anhydrous DCM (5.0 mL), trifluoroacetic acid (2.5 mL) was added dropwise. The resulting mixture was stirred at room temperature for 19 h, after which the solvent was removed *in vacuo* to give the compound **S2** as a white solid (0.061 g, quant.). ^{1}H NMR (400 MHz, d_6-acetone) δ 7.49 (s, 2H), 3.70 (s, 2H).

Amidation

General procedure for EDC-mediated coupling (A). Carboxylic acid **S2** (0.30 mmol), amine (0.45 mmol, 1.5 equiv), HOBt hydrate (0.45 mmol, 1.5 equiv), and EDC·HCl (0.45 mmol, 1.5 equiv) were dissolved in anhydrous DCM (3 mL). The mixture was irradiated under microwave irradiation at 60 °C for 2 h, after which it was diluted with DCM (10 mL). The solution was washed with a saturated solution of NH_4Cl in H_2O, water, and brine. The organic phase was dried over anhydrous Na_2SO_4, filtered, and concentrated *in vacuo*. The crude product was purified with automated flash column chromatography (*n*-heptane/EtOAc-EtOH 3:1 (12→100%) to give the pure product.

General procedure for EDC-mediated coupling (B). Carboxylic acid **S2** (0.3 mmol) was dissolved in anhydrous DCM and anhydrous THF at 0 °C. HOBt (0.1 equiv), and EDC·HCl (1.1 equiv) were added and the mixture was stirred at 0 °C for 15 min. After this, the amine (1.2 equiv) was added, and the reaction mixture was stirred at room temperature for 19 h. The solvent was removed *in vacuo* (compounds **66–67**, **70–71**) or diluted with DCM (10 ml), washed with saturated solution of $NaHCO_3$ in H_2O (5 mL) and brine (5 mL), dried over Na_2SO_4, filtered before removal of solvent *in vacuo* (compounds **68–69**). The crude product was purified with automated flash column chromatography (*n*-heptane/EtOAc gradient 0→100%) to give the pure product. Procedure B for compounds **66–71**.

7,9-Dichloro-*N*-(3-chloro-4-methoxyphenyl)-8-oxo-1-oxa-2-azaspiro[4.5]deca-2,6,9-triene-3-carboxamide (65)

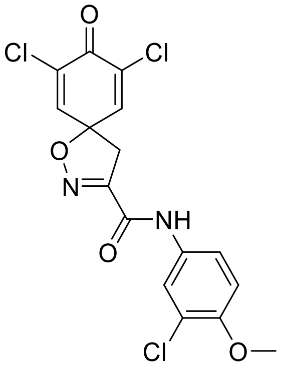

General procedure A was followed to give the compound **65** as light yellow solid (0.022 g, 16%). ^{1}H NMR (400 MHz, d_6-acetone) δ 9.52 (br s, 1H), 7.97 (d, J = 2.6 Hz, 1H), 7.72 (ddd, J = 9.0, 2.6, 1.9 Hz, 1H), 7.52 (d, J = 0.8 Hz, 2H), 7.12 (d, J = 9.0 Hz, 1H), 3.90 (s, 3H), 3.75 (s, 2H). ^{13}C NMR (101 MHz, d_6-acetone) δ 172.6, 157.6, 156.1, 152.9, 142.5, 132.6, 122.9, 122.5, 120.8, 113.5, 85.5, 68.1, 56.7, 44.1. HRMS (ESI+): calculated 399.9784 ($C_{16}H_{11}Cl_3N_2O_4$), found 399.9786. LC-MS: $[M + H]^+$ m/z 402 (t_r = 4.80 min), >95%.

7,9-Dichloro-8-oxo-*N*-[2-(pyridin-2-yl)ethyl]-1-oxa-2-azaspiro[4.5]deca-2,6,9-triene-3-carboxamide (66)

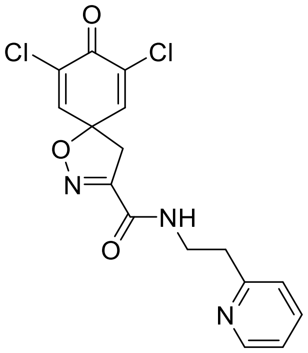

General procedure B was followed to give the compound **66** as white solid (0.076 g, 55%). ^{1}H NMR (400 MHz, CD_3CN) δ 8.51 (ddd, J = 4.9, 1.9, 1.0 Hz, 1H), 7.69 (td, J = 7.7, 1.9 Hz, 1H), 7.59 (br s, 1H), 7.27-7.18 (m, 4H), 3.65 (td, J = 6.8, 5.9 Hz, 2H), 3.47 (s, 2H), 3.00 (t, J = 6.8 Hz, 2H). ^{13}C NMR (101 MHz, CD_3CN) δ 173.2, 160.2, 159.2, 155.8, 150.0, 142.5, 137.7, 132.7, 124.4, 122.7, 85.0, 44.2, 39.6, 37.5. HRMS (ESI+): calculated 366.0412 ($C_{16}H_{14}Cl_2N_3O_3$), found 366.0415. LC-MS: $[M + H]^+$ m/z 366 (t_r = 1.88 min), >95%.

7,9-Dichloro-8-oxo-*N*-[2-(pyridin-3-yl)ethyl]-1-oxa-2-azaspiro[4.5]deca-2,6,9-triene-3-carboxamide (67)

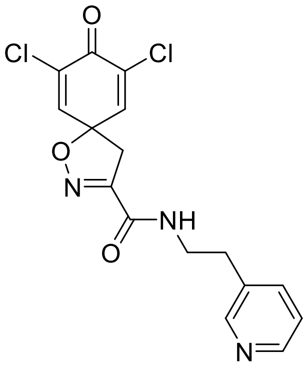

General procedure B was followed to give the compound **67** as white solid (0.070 g, 63%). ^{1}H NMR (400 MHz, CD_3CN) δ 8.46 (dd, J = 2.3, 0.8 Hz, 1H), 8.43 (dd, J = 4.8, 1.7 Hz, 1H), 7.62 (m, 1H), 7.28 (ddd, J = 7.8, 4.8, 0.9 Hz, 1H'), 7.23 (s, 2H), 7.22 (br s, 1H), 3.53 (td, J = 7.0, 6.2 Hz, 2H), 3.45 (s, 2H), 2.86 (t, J = 7.1 Hz, 2H). ^{13}C NMR (101 MHz, CD_3CN) δ 173.2, 159.4, 155.7, 151.2, 148.7, 142.5, 137.2, 135.6, 132.7, 124.3, 85.0, 44.1, 41.0, 33.2. HRMS (ESI+): calculated 366.0412 ($C_{16}H_{14}Cl_2N_3O_3$), found 366.0415. LC-MS: $[M + H]^+$ m/z 366 (t_r = 1.85 min), >99%.

7,9-Dichloro-8-oxo-*N*-(pyridin-3-yl)-1-oxa-2-azaspiro[4.5]deca-2,6,9-triene-3-carboxamide (68)

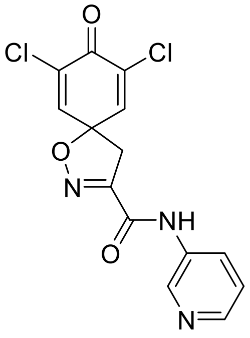

General procedure B was followed to give the compound **68** as yellow solid (0.032 g, 35%). ^{1}H NMR (400 MHz, CD_3CN) δ 8.98 (s, 1H), 8.81 (dd, *J* = 2.7, 0.8 Hz, 1H), 8.37 (dd, *J* = 4.7, 1.5 Hz, 1H), 8.09 (ddd, *J* = 8.3, 2.6, 1.5 Hz, 1H), 7.36 (m, 1H), 7.28 (s, 2H), 3.57 (s, 2H). ^{13}C NMR (101 MHz, CD_3CN) δ 173.2, 158.3, 155.9, 146.8, 143.1, 142.2, 135.2, 133.0, 128.5, 124.6, 85.7, 43.8. HRMS (ESI+): calculated 338.0099 ($C_{14}H_{10}Cl_2N_3O_3$), found 338.0102. LC-MS: $[M + H]^+$ *m*/*z* 338 (t_r = 2.20 min), >96%.

7,9-Dichloro-8-oxo-*N*-(pyridin-2-yl)-1-oxa-2-azaspiro[4.5]deca-2,6,9-triene-3-carboxamide (69)

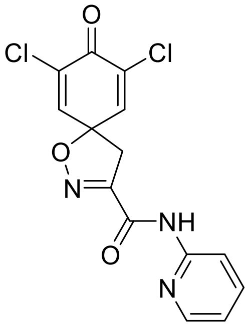

General procedure B was followed to give the compound **69** as white solid (0.022 g, 21%). ^{1}H NMR (400 MHz, CD_3CN) δ 8.94 (s, 1H), 8.35 (ddd, J = 4.9, 1.9, 0.9 Hz, 1H), 8.11 (dt, *J* = 8.4, 1.0 Hz, 1H), 7.82 (ddd, *J* = 8.3, 7.4, 1.9 Hz, 1H), 7.28 (s, 2H), 7.17 (ddd, *J* = 7.4, 4.9, 1.0 Hz, 1H), 3.57 (s, 2H). ^{13}C NMR (101 MHz, CD_3CN) δ 173.2, 158.0, 156.0, 151.4, 149.5, 142.1, 139.6, 133.0, 121.6, 114.9, 86.0, 43.5. HRMS (ESI+): calculated 338.0099 ($C_{14}H_{10}Cl_2N_3O_3$), found 338.0098. LC-MS: $[M + H]^+$ *m*/*z* 338 (t_r = 3.69 min), >96%.

***N*-(3-Bromo-4-methoxyphenyl)-7,9-dichloro-8-oxo-1-oxa-2-azaspiro[4.5]deca-2,6,9-triene-3-carboxamide (70)**

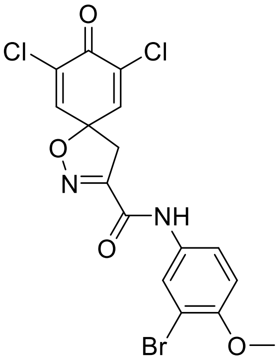

General procedure B was followed to give the compound **70** as yellow solid (0.083 g, 49%). ^{1}H NMR (400 MHz, d_6-DMSO) δ 10.61 (s, 1H), 8.05 (d, *J* = 2.5 Hz, 1H), 7.73 (dd, *J* = 9.0, 2.6 Hz, 1H), 7.64 (s, 2H, H), 7.13 (d, *J* = 9.0 Hz, 1H,), 3.84 (s, 3H), 3.67 (s, 2H). ^{13}C NMR (101 MHz, d_6-DMSO) δ 171.9, 156.8, 155.4,

152.1, 142.2, 132.0, 130.8, 124.8, 120.9, 112.6, 110.0, 84.1, 56.3, 43.4. HRMS (ESI+): calculated 446.9355($C_{16}H_{12}BrCl_2N_2O_4$), found 446.9318. LC-MS: $[M + H]^+$ *m/z* 447 (t_r = 4.92 min), >96%.

7,9-Dichloro-*N,N*-dimethyl-8-oxo-1-oxa-2-azaspiro[4.5]deca-2,6,9-triene-3-carboxamide (71)

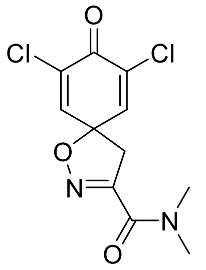

General procedure B was followed to give the compound **71** as white solid (0.041 g, 34%). ^1H NMR (400 MHz, $CDCl_3$) δ 7.05 (s, 2H), 3.56 (s, 2H), 3.32 (s, 3H), 3.09 (s, 3H). ^{13}C NMR (101 MHz, $CDCl_3$) δ 172.0, 159.2, 153.7, 140.5, 133.2, 82.4, 46.2, 38.8, 36.4. HRMS (ESI+): calculated 289.0147 ($C_{11}H_{11}Cl_2N_2O_3$), found 289.0150. LC-MS: $[M + H]^+$ *m/z* 289 (t_r = 3.10 min), >99%.

7,9-Dichloro-8-oxo-*N*-(pyridin-3-ylmethyl)-1-oxa-2-azaspiro[4.5]deca-2,6,9-triene-3-carboxamide (72)

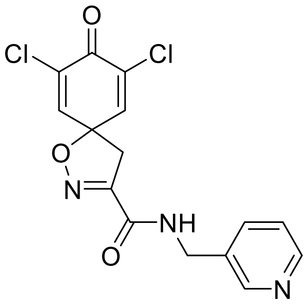

General procedure A was followed to give the compound **72** as white solid (0.010 g, 13%). ^1H NMR (400 MHz, d_6-acetone) δ 8.62–8.56 (m, 1H), 8.48 (dd, *J* = 4.9, 1.6 Hz, 1H), 8.37 (s, 1H), 7.75 (ddd, *J* = 7.8, 2.3, 1.7 Hz, 1H), 7.49 (s, 2H), 7.32 (ddd, *J* = 7.8, 4.8, 0.9 Hz, 1H), 4.55 (d, *J* = 6.3 Hz, 2H), 3.69 (s, 2H). ^{13}C NMR (101 MHz, d_6-acetone) δ 172.6, 159.5, 155.7, 150.3, 149.4, 142.7, 136.2, 135.3, 132.5, 124.2, 85.1, 44.3, 41.3. HRMS (ESI+): calculated 352.0256 ($C_{15}H_{12}Cl_2N_3O_3$), found 352.0258. LC-MS: $[M + H]^+$ *m/z* 352 (t_r = 1.84 min), >98%.

Figure S1. Reversibility of binding of compounds 74, 76, 79

Normalized Cav3.2 channel current during wash-in and wash-out of compound 74, 76 and 79 over time.

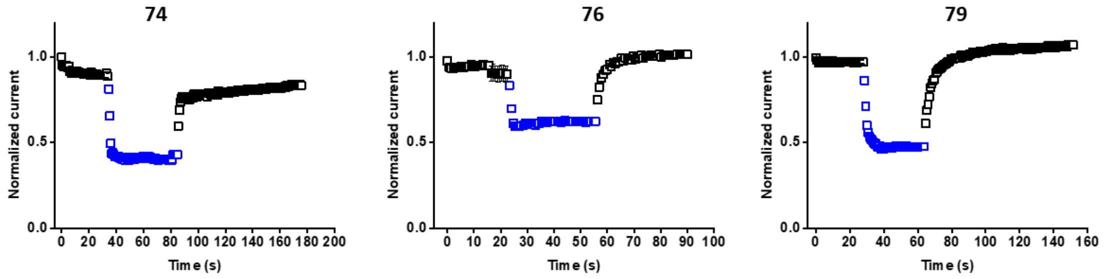

Supplement: Supplementary file 1 [file ijms-24-03429-s001.zip › ijms-2165040-supplementary.pdf]
